# Advanced Strategies for Overcoming Endosomal/Lysosomal Barrier in Nanodrug Delivery

**DOI:** 10.34133/research.0148

**Published:** 2023-05-24

**Authors:** Chong Qiu, Fei Xia, Junzhe Zhang, Qiaoli Shi, Yuqing Meng, Chen Wang, Huanhuan Pang, Liwei Gu, Chengchao Xu, Qiuyan Guo, Jigang Wang

**Affiliations:** ^1^ Artemisinin Research Center, and Institute of Chinese Materia Medica, China Academy of Chinese Medical Sciences, Beijing 100700, China.; ^2^ Department of Nephrology, and Shenzhen Clinical Research Centre for Geriatrics, Shenzhen People's Hospital, The First Affiliated Hospital, Southern University of Science and Technology, Shenzhen, Guangdong 518020, China.

## Abstract

Nanocarriers have therapeutic potential to facilitate drug delivery, including biological agents, small-molecule drugs, and nucleic acids. However, their efficiency is limited by several factors; among which, endosomal/lysosomal degradation after endocytosis is the most important. This review summarizes advanced strategies for overcoming endosomal/lysosomal barriers to efficient nanodrug delivery based on the perspective of cellular uptake and intracellular transport mechanisms. These strategies include promoting endosomal/lysosomal escape, using non-endocytic methods of delivery to directly cross the cell membrane to evade endosomes/lysosomes and making a detour pathway to evade endosomes/lysosomes. On the basis of the findings of this review, we proposed several promising strategies for overcoming endosomal/lysosomal barriers through the smarter and more efficient design of nanodrug delivery systems for future clinical applications.

## Introduction

Owing to their unique physicochemical, biological, optical, electrical, and catalytic activities, nanodrugs can overcome the pharmacokinetic limitations associated with traditional pharmaceutical agents [1,2]. An intravenously delivered nanodrug typically has to pass through 5 consecutive processes [3]: circulation in the blood compartments, accumulation into the target area, subsequent penetration deeply into the tissue, cellular uptake by cells, and intracellular release of drug from endosome or lysosome. Therefore, researchers have designed nanocarriers with different assembly modes to overcome multiple intracellular obstacles during delivery [4]. Because most drug delivery systems pass through the endosome–lysosome pathway after cellular uptake, the ability of endosomal/lysosomal escape is one of the crucial factors affecting delivery efficiency [5]. Most intracellular transport pathways include internalization into an endocytic vesicle, fusion with the early endosome (EE), maturation into a late endosome (LE), and accumulation in the lysosome [6]. Significantly, compared with the physiological pH of 7.4, the pH gradually decreases during maturation of the endosome (EE pH ∼ 6.5, LE pH ∼ 6.0, and lysosome has a lower pH ∼ 5.0) [7]. Lysosomes contain various degradative enzymes (such as nucleases and phosphatases), and failure to escape rapidly from lysosomes usually results in entrapment and potential degradation, leading to the unsuccessful delivery of therapeutic drugs [8,9].

Overcoming the endosomal/lysosomal barrier is a crucial step in the successful delivery of nanodrugs for the treatment of various diseases, including cancer, neurodegenerative disorders, and infectious diseases. For example, the blood–brain barrier (BBB) can prevent nanodrugs from reaching the brain, making it challenging to treat brain glioma and neurodegenerative disorders such as Alzheimer’s disease and Parkinson’s disease [10]. Most nanocarriers undergo lysosomal degradation after they are internalized by endothelial cells via endocytosis, failing to penetrate the BBB via transcytosis. Strategies to overcome the endosomal/lysosomal barrier are helpful to deliver therapeutic agents across BBB. Similarly, strategies such as pH-sensitive or fusogenic nanoparticles can help release drugs from the endosomal/lysosomal compartments of infected cells, improving their efficacy to treat hepatitis B and C viruses.

To achieve rapid drug release into the cytoplasm, various strategies have been reported in research articles and reviews. These strategies are mainly related to various biological mechanisms [11–16], including the proton sponge effect, membrane instability, membrane fusion, and membrane disruption. However, most previous reviews have primarily focused on strategies that promote lysosomal escape, thus limiting the findings, instead of summarizing more strategies from the perspective of cellular and intracellular transport mechanisms. For example, strategies that do not involve entry into endosomes or lysosomes have not been considered in some reviews [17]. To this end, this review highlights the following 3 strategies for overcoming lysosomal barriers based on the perspective of cellular uptake and intracellular transport mechanisms: (a) promoting endosomal/lysosomal escape, (b) crossing the cell membrane without entering endosomes or lysosomes, and (c) making a detour pathway to evade lysosomes and avoid degradation. These strategies may provide novel and more comprehensive design ideas for overcoming endosomal/lysosomal barriers to efficient nanodrug delivery.

## Cellular Uptake and Intracellular Transport Mechanisms

An in-depth understanding of the mechanisms of cellular and intracellular transport is necessary to resolve the problem of endosomal/lysosomal degradation. Although studies on intracellular transport and lysosomal escape are limited [18], some studies have comprehensively investigated the molecular mechanisms underlying these processes [9,19,20]. For example, Gilleron et al. [9] used fluorescence imaging and electron microscopy to examine the cellular uptake and delivery characteristics of an ionizable lipid nanoparticle-based delivery system. This system mainly entered the cell through clathrin-mediated endocytosis (CME) and micropinocytosis (MP); however, only a small fraction of small interfering RNA (siRNA) (<2%) was released into the cytoplasm from endosomes/lysosomes. Similarly, another study reported that approximately 70% of siRNA was transported to the extracellular medium by circulating endosomes through a pathway mediated by Niemann–Pick C1 within 24 h of nanoparticle uptake [21]. Fichter et al. [20] indicated that Glycofect polymer nanoparticles were delivered via a non-sibling internal transport pathway, which induced their higher accumulation in the Golgi apparatus and endoplasmic reticulum (ER) owing to the presence of galactose in the nanoparticles. Therefore, the intracellular transport of drugs can be achieved by not only the endosome–lysosome pathway but also the different transport routes that may help to overcome endosomal/lysosomal barriers. However, the mechanisms underlying the internalization of extracellular materials and their intracellular transport vary greatly depending on the type of materials [22]. It is necessary to understand these mechanisms to design better delivery vectors and resolve the subsequent problem of endosomal/lysosomal degradation.

### Cellular uptake mechanisms

**Fig. 1.**
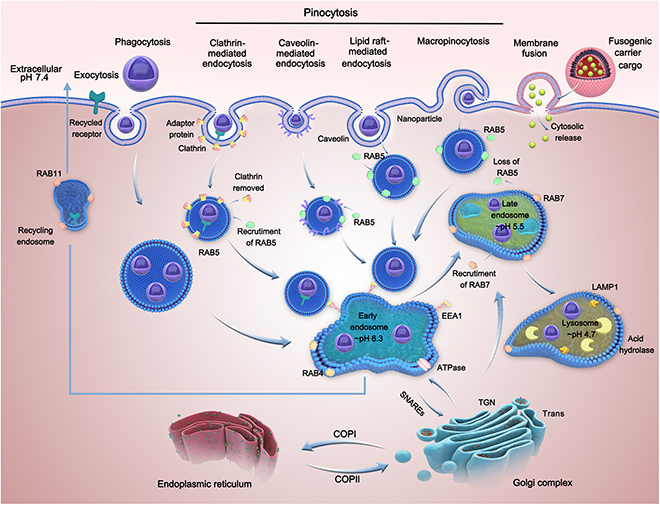
Routes and mechanisms of cellular uptake and intracellular transport. ATPase, adenosine triphosphatase.

Cellular uptake, intracellular transport, and localization of nanoparticles are primarily determined by the physical and chemical properties of nanocarriers, including their size, zeta potential, and surface modification [23]. On the basis of their size and surface modification, nanoparticles are internalized through phagocytosis or pinocytosis. Phagocytosis refers to the internalization of bacteria, fragments, or cargos of large sizes, whereas pinocytosis refers to the internalization of smaller components (Fig. 1). Endocytic pathways can be subdivided into CME, caveolae-mediated endocytosis (CvME), lipid raft-mediated endocytosis, MP, and membrane fusion [24,25].

CME is regulated by clathrin and its adaptor proteins. Pentagonal or hexagonal grid-like structural concavities initially form capsule vesicles, and nanoparticles are endocytosed through several processes as follows: recruitment of an adaptor protein and clathrin, invagination and constriction of capsule vesicles, budding of vacuoles, and shelling of coated vacuoles [26]. CME is an important cellular uptake pathway for endogenous substances, including transferrin and low-density lipoprotein. Studies have demonstrated that a drug delivery system modified with endogenous substances is mainly internalized through CME [27]. For example, nanoparticles modified with peptide-22 targeting low-density lipoprotein enter cells mainly through reticulin-mediated endocytosis [28]. In addition, studies have demonstrated that most delivery systems designed based on lipids are internalized via CME [29].

CvME is coordinated by caveolae, which are cup-shaped/omega-shaped invaginations of the plasma membrane. Lipid density, cholesterol content, glycosphingolipid content, and sphingomyelin content are relatively high in the bilayer structure [30]. In addition to dynein, glycosylphosphatidylinositol-anchored proteins and tyrosine kinases, caveolin, and caveolae-related proteins are characteristic components of caveolae. Although the specific regulatory mechanisms remain unclear, many nanocarriers can enter the cell via CvME, such as magnetic nanoparticles containing siRNA of green fluorescent protein [31]. In addition, the uptake pathway of the polyethyleneimine (PEI)/DNA and poly(amidoamine) (PAMAM)/DNA complexes is associated with caveolin-mediated endocytosis [32]. Note that the diameter of caveolin vesicles is approximately 30 to 80 nm, and nanoparticles with a large particle size cannot be internalized through CvME. For example, silica nanoparticles with a 90-mm diameter modified with antibodies targeting HER2 can enter the cells through CvME; however, nanoparticles with a 200-nm diameter cannot be internalized through CvME [33]. In addition, surface properties can affect endocytosis. For example, a high-glucose environment can inhibit reticulin-mediated endocytosis and enhance CvME. Park et al. [34] prepared the polymannose PEI material (PMT) by cross-linking PEI with mannose diacrylate, and the results indicated that the PMT/DNA complex was internalized via caveolin-mediated endocytosis.

Similar to caveolae, lipid rafts are microregions on the cell membrane that are rich in protein receptors and sphingolipids and are highly ordered and more compact. Viral particles are internalized through lipid raft-induced endocytosis through glycoprotein binding [35], and low-density lipoprotein can promote the endocytosis of the amyloid precursor protein through this pathway [36].

MP is a method of nonselective endocytosis of water-soluble molecules [37]. It involves the formation of large and irregular endocytic vesicles derived from the cell membrane and mediates the endocytosis of nanoparticles with large particle sizes (250 nm) [38]. It depends on actin and is regulated by various proteins, such as phosphoinositide 3 kinase and Rab family members. Additionally, MP is sensitive to the pH of the cytoplasm. Cytoplasmic acidification can significantly reduce the movement of cell membrane folds [39]. Some lipid-based nucleic acid delivery systems are thought to enter cells through macrocytosis. For example, macrocytosis is considered the only pathway through which “core–shell” nanocarriers constructed on the basis of protamine/DNA/cationic liposomes are internalized by Chinese hamster ovary (CHO) cells [40].

Another mechanism of cell uptake is direct fusion with the cell membrane, which successively goes through lipid bilayers close to each other, external hydrophilic layer direct contact, protein-mediated fusion, and finally the fusion pore opens. This mechanism is mediated by different proteins, mainly including SNAREs, (soluble N -ethylmaleimide-sensitive factor attachment protein receptors), Rab proteins, and Sec1/Munc-18 related proteins [41,42]. Exosomes, biofilm carriers, and membrane-penetrating peptide-modified carriers are often internalized via membrane fusion because they have a corresponding affinity for target cell membranes, which renders membrane fusion more efficient. For example, Yang et al. [43] prepared virus-like exosomes that could directly deliver the encapsulated protein into target cells through membrane fusion.

### Intracellular transport mechanisms

After nanoparticles are endocytosed through the abovementioned pathways, they are transported intracellularly through different pathways involving endosomes, lysosomes, the Golgi apparatus, and ER, owing to the differences in their cellular uptake pathways and particle characteristics. To date, 3 intracellular transport pathways have been widely reported, namely, the classical endosome–lysosome pathway, the endosome–Golgi pathway, and the Golgi–ER pathway.

The endosome–lysosome pathway is the most common intracellular transport mechanism, which is closely related to the maturation of endosomes. As shown in Fig. 1, primary endocytic vesicles deliver their contents to EEs around the cytoplasm. After approximately 8 to 15 min, the contents accumulate in EEs and are recycled to the plasma membrane (directly or through internal circulation of endosomes), resulting in the transformation of EEs to LEs. As LEs move along the microtubule (MT) to the periphery of the nucleus, they fuse with newly formed lysosomes and transform into the classic mature lysosomes with a low pH [44]. Consequently, when nanocarriers are transported through the endosome–lysosome pathway, they are bound to undergo degradation and destruction by acids and enzymes, which is detrimental to the subsequent effects of drugs. Therefore, the endosome–lysosome pathway is considered a degradative intracellular transport pathway. Studies have demonstrated that nanocarriers that are internalized by cells through CME or part of MP are transported intracellularly through the endosome–lysosome pathway. For example, the liposome/VEGF (vascular endothelial growth factor) siRNA system modified with angiopep and tLyP-1 peptides enters lysosomes after its internalization through CME [45]. Similarly, core–shell nanoparticles, which enter cells through macrocytosis, rapidly colocalize with lysosomes in the cells [40].

The Golgi apparatus acts as a “transit station” for intracellular transport (Fig. 1). After endocytosis, the internalized contents are initially transported to the Golgi apparatus, which generates new vesicles in the form of “budding.” These vesicles are transported to endosomes, lysosomes, or ER via regulation of various signals. The transport from endosomes to the Golgi apparatus is regulated by acid hydrolase receptors, transmembrane enzymes, and SNARE proteins, which can initiate retrograde transport and fusion from EEs/LEs to the trans-Golgi network (TGN) [46]. This transport pathway is considered an important mechanism to avoid lysosomal degradation after endocytosis [47]. SNAREs are membrane proteins that are widely distributed in the ER, Golgi apparatus, and endosomes and are sparsely distributed on the cell membrane. According to the SNARE hypothesis, v-SNARE on transport vesicles specifically interacts with homologous t-SNARE on the target membrane, and the formation of a very stable 4-helix bundle results in the fusion of the 2 diaphragms [47]. For example, shiga and cholera toxins are transported from endosomes to TGN and eventually to ER, where they exert their cytotoxic effects [48].

The 2 types of transport modes between the Golgi apparatus and ER are as follows: (a) COPI vesicles mediate the transport from the Golgi apparatus to ER, and (b) COPII vesicles mediate the transport from the ER to Golgi apparatus. Retrograde transport by COPI vesicles mainly involves various types of proteins [49], with the most important proteins being resident proteins with a representative KDEL signal (Lys-Asp-Glu-Leu tetrapeptide). The KDEL receptor interacts with soluble secreted proteins at the lower pH of the Golgi apparatus and is directed to ER through COPI vesicles. Subsequently, the receptor releases resident proteins into the cavity at the neutral pH of ER. COPI vesicles can induce transport from the Golgi apparatus to ER, thereby avoiding lysosomal degradation. Therefore, the Golgi–ER pathway is considered a non-degradative transport mode for overcoming lysosomal barriers.

## Strategies for Overcoming Endosomal/Lysosomal Barriers

**Fig. 2.**
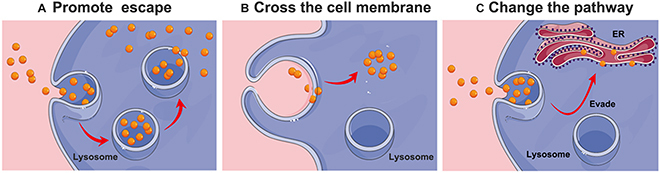
A schematic overview of 3 main strategies used for overcoming lysosomal barriers. (A) Strategies to promote endosomal/lysosomal escape. (B) Strategies for directly crossing the cell membrane without entering endosomes or lysosomes. (C) Strategies to change the pathway to evade lysosomes and avoid degradation.

On the basis of the abovementioned mechanisms of cellular uptake and intracellular transport, researchers have developed several strategies for resolving the problem of lysosomal degradation and destruction (Fig. 2). These strategies are primarily divided into 3 categories as follows: (a) strategies for promoting endosomal/lysosomal escape, (b) strategies for directly crossing the cell membrane without entering endosomes or lysosomes, and (c) strategies in which different pathways are used to evade lysosomes and avoid degradation. Furthermore, these strategies for overcoming endo-/lysosomal barrier in nanodrug delivery have been summarized in Table.

**Table.** The summary of strategies for overcoming endo/lysosomal barrier in nanodrug delivery.

Strategy	Mechanism	Materials	Drug/agent	Disease/cells	Ref.
**Promote endo/lysosomal escape**	**Proton sponge effect**	Poly-l-lysine (PLL), polyphenol (-)-epigallocatechin gallate (EGCG)	dsRNA	Sf9 cells	[50]
		Poly(amidoamine) dendrimers (PAMAM)	Cisplatin	BxPC-3 cells	[51]
		Poly(histidine-arginine)_6_-modified chitosan	siRNA	4T1 cells and B16F10 cells	[52]
		PEG-PCL-PEI	siRNA	SKOV3 cells	[53]
		Pyridinium p-toluenesulfonate (PPTS)	Doxorubicin	Panc-1 cells	[54]
	**Osmotic lysis effect**	PMPC-bPDPAEMA	Dyes	22 different cells	[55]
		mPEG_45_-P(DPA_50_-co-DMAEMA_56_)-PT_53_	siRNA	HepG2 cells	[56]
		DOPA, DOTAP, CaP nanoparticles (NPs)	siRNA	H460 cells	[57,58]
		Alendronate-hyaluronan, CaP NPs	siRNA	A549 cells	[59]
	**Swelling effect**	PDEAEMA, PAEMA, PEGDMA	Ovalbumin	DC2.4 cells	[60]
		2,4,6-Trimethoxy benzaldehyde, 5-methyl-2-(2,4,6-trimethoxy phenyl)-[1,3]-5-dioxanylmethanol	Paclitaxel	LLC cells	[61]
		Poly(c-GA-co-c-GAOSu)-g-mPEG	Doxorubicin	HeLa cells	[62]
	**Pore formation**	LPEI polyplexes	ODA/pDNA	HeLa cells	[63]
		PEI, HIV gp41 peptide	siRNA/pDNA	HeLa cells	[64]
		PEGylated Au-NPs	LLO protein	MEF cells	[65]
	**Membrane disruption**	Aptamer modified iron-deficient protein nanocages (Apn), HA-2 peptide	siRNA	MCF-7 cells	[66]
		DSPE-PCB lipoplexes	siRNA	HeLa cells	[67]
		Ultrasound-responsive polymersome PEO-b-P(DEA-stat-MEMA)	Doxorubicin	L02 cells and HeLa cells	[68]
		Temperature-sensitive “bubble liposomes”: PEG modified DOTAP/DOPE/cholesterol liposomes, ammonium bicarbonate	siRNA	H69AR cells	[69]
	**Membrane fusion**	Core–shell liposomes: DSPE-PEG/DOTAP/DOPE/cholesterol	siRNA	U87 and MCF-7 cells	[70–73]
		R8/KALA-T-MEND	pDNA	JAWS II cells	[74]
		PEgylated Tf-liposome incorporating Chol-GALA	pDNA	K562 cells	[75]
	**Photochemical internalization**	CPP-cargo-linker-photosensitizer	shRNA	CHO cells	[76]
		TPPS2A, silica core–shell NPs	Photomorpholinos	B16-F0 cells	[77]
		thiolated CPT (CPT-DP), thiolated PEG-b-P(Glu-DP)	Camptothecin (CPT)	HeLa cells	[78]
**Non-endocytosis delivery by directly crossing the cell membrane**	**Membrane fusion**	PEG/fusogenic porous silicon NPs	siRNA	CAOV-3 cells	[79]
		F68, PCL, cancer cell membrane	Paclitaxel	4T1 cells	[80]
		Coiled-coil SNARE lipopeptides (CPK_4_, CPE_4_)	Doxorubicin	HeLa cells	[81]
		Oligonucleotide (ON) template-assisted polymerization	Oligonucleotides	HeLa cells	[82]
		CPP/single-wall carbon nanotubes	siRNA	HeLa cells	[83]
		Carbon nanotube porins (CNTPs)	Doxorubicin	MDA-MB231 cells	[84]
**Make a detour pathway to evade lysosomes**	**Cavosome-mediated transport**	CPG-modified siRNA	siRNA	RAW264.7 and B16 cells	[85]
		Histone/PEI polyplexes	pDNA	CHO cells	[86]
		Transferrin-PEI/folate-PEI	siRNA	HeLa cells	[87]
	**Transported to Golgi/ER**	KDEL modified Au NPs	pDNA	Sol8 cells	[88]
		ER membrane decorated DOTAP/DOPE/cholesterol liposome	siRNA	MCF-7 cells	[89]

### Endosomal/lysosomal escape

**Fig. 3.**
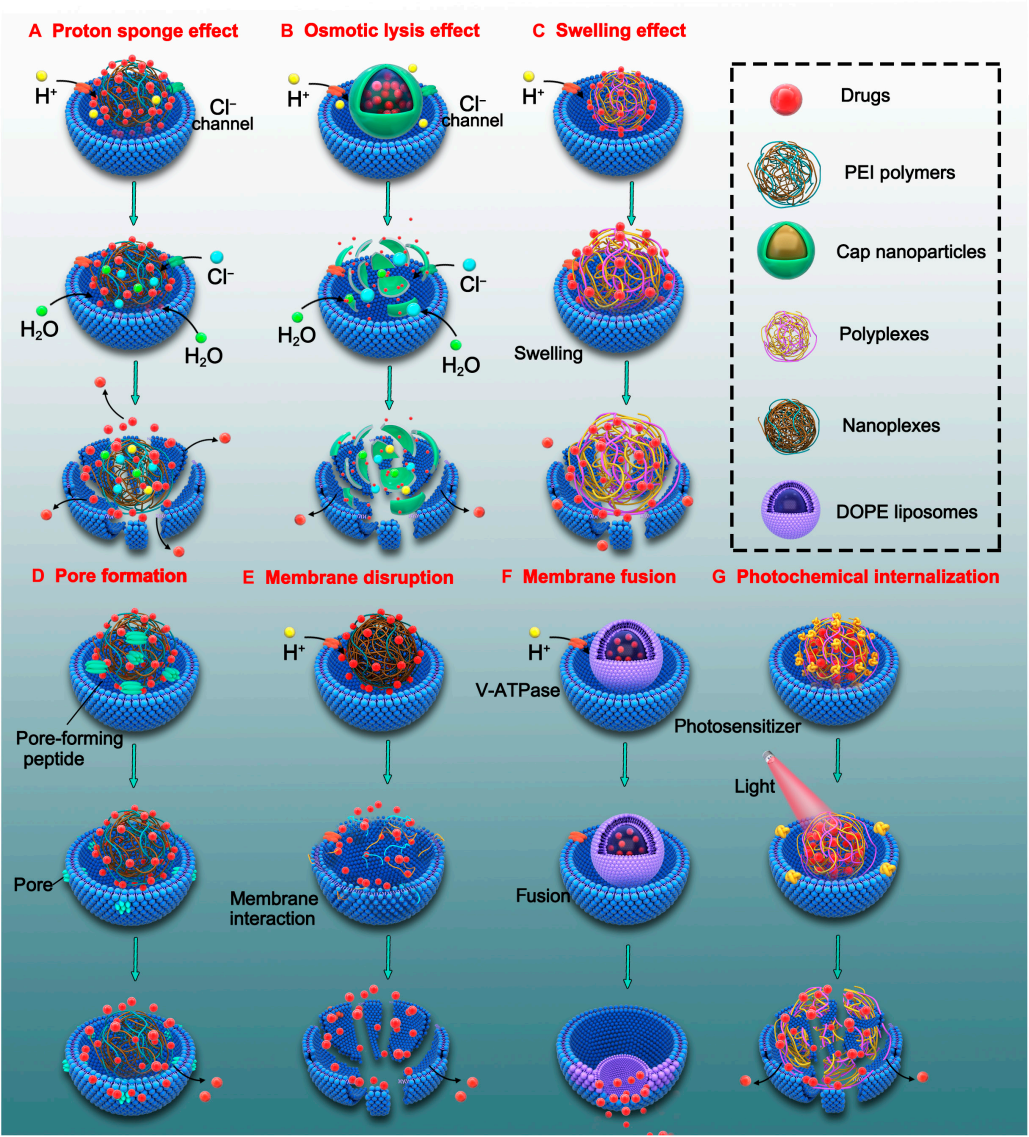
(A to G) A schematic overview of mechanisms underlying endosomal escape.

The endosome–lysosome pathway is considered a degradative intracellular transport pathway because nanoparticles are bound to undergo degradation and destruction by acids and enzymes, which is detrimental to the subsequent effects of encapsulated drugs. Therefore, the first goal is to escape from endosomes or lysosomes rapidly. The mechanisms underlying endosomal/lysosomal escape are highly controversial but involve trafficking across the endosomal/lysosomal membrane (Fig. 3).

This section describes 4 advanced strategies used for promoting endosomal/lysosomal escape of nanodrugs: the proton sponge effect of outstanding pH buffering; osmotic lysis resulting from pH-responsive disassembly of nanoparticles; as well as the swelling effect of pH-responsive nanoparticles and membrane destabilization induced by pore formation, membrane disruption, membrane fusion, and photochemical internalization.

#### Endosomal/lysosomal escape through the proton sponge effect

**Fig. 4.**
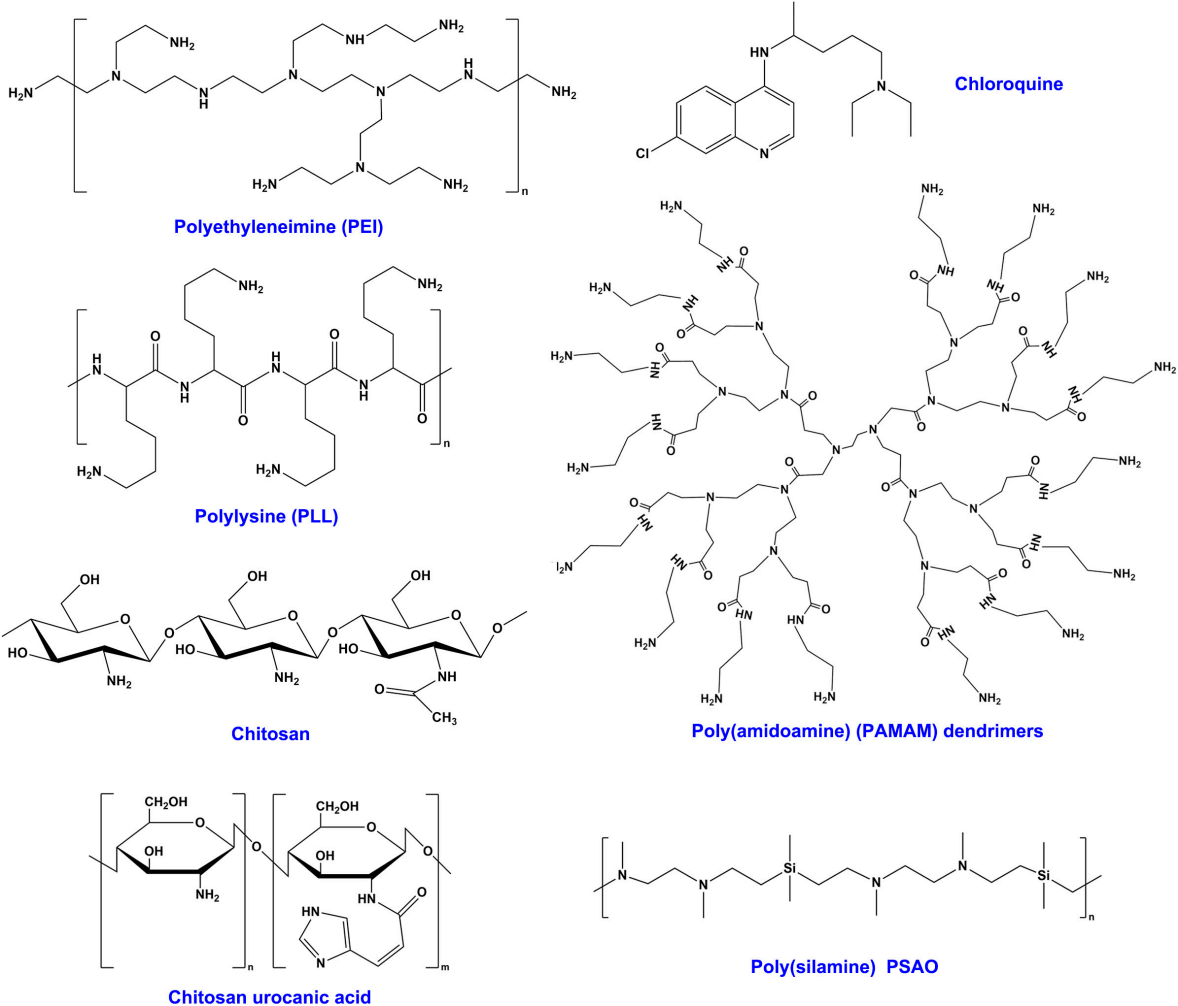
Structure of representative materials with the capability to escape from endosomes or lysosomes via the proton sponge effect.

The proton sponge effect is a typical method of inducing endosomal/lysosomal escape based on the buffering action of materials in a physiologically relevant range (Fig. 3A) [90]. The protonated amino groups in cationic materials are chelated with protons provided using proton pumps (vacuolar-ATPase), resulting in the continuous opening of the pumps. Each proton can result in the entrapment of a chloride ion and water molecule in lysosomes, thereby leading to the swelling and rupture of lysosomes and the release of cationic nanocarriers into the cytoplasm. This strategy is usually applied to polycationic materials, including PEI [91], poly-l-lysine (PLL) [50,92,93], PAMAM dendrimers [94], chitosan [95], poly(silamine) [96], urocanic acid-modified chitosan [97], chloroquine [98], and others containing secondary or tertiary amines (Fig. 4).

Owing to the inclusion of protonatable amino groups, the abovementioned materials are protonated to produce a “proton sponge” effect at the low pH (4 to 5.5) of endosomes or lysosomes. For example, Sun et al. [52] covalently linked poly(histidine arginine) (H_6_R_6_) with chitosan to encapsulate siRNAs, and the lysosomal escape ability of these modified chitosan nanoparticles was significantly stronger than that of unmodified chitosan nanoparticles. On the basis of the proton sponge effect, materials have been designed as a component of delivery systems for encapsulating drugs and facilitating their lysosomal escape, such as the triblock copolymers PEG-PCL-PEI for delivering siRNA [53] and PAMAM/carbon dot nanohybrids for chemotherapy [99]. Lee et al. [100] prepared rod-shaped nanoparticles formed by bundles of Janus base nanotubes (JBNTs) with RNA cargos incorporated inside via charge interactions. Similar to lipid nanoparticles, JBNTs/RNA nanoparticles efficiently entered cells via macrocytosis. Additionally, similar to cationic polymers, JBNTs/RNA nanoparticles had an enhanced endosomal escape ability because of their noncovalent structure and DNA-mimicking chemistry, which promoted the proton sponge effect. Chen et al. [12] developed metal-phenolic networks assembled with the polyphenol tannic acid and Fe_III_ or Al_III_ as versatile and nontoxic modifications to promote endosomal/lysosomal escape through the proton sponge effect. The lysosomal escape ability was closely related to the amount of material used [101]. Effective transfection necessitates a significant excess of polymers, which frequently causes dose-limiting toxicity during in vivo application [14].

With the understanding and development of the proton sponge theory, researchers have developed various polymeric nanocarriers with increased buffering capacities. However, these nanocarriers have limited endosomal escape ability. Studies have reported that the buffering capacity of some polymer-based delivery systems was insufficient to achieve endosomal escape during their transport from endosomes/lysosomes to the cytoplsm [54,102]. Because only a few lysosomes per cell can fluctuate (a few hundred to several thousand lysosomes are found in each cell), only 1% of carriers can achieve escape [103,104]. Benjaminsen et al. [104] demonstrated that polyplexes prepared using PEI or derivatives did not alter the endosomal/lysosomal pH and suggested that the proton sponge effect did not play a vital role in escape. However, these results are based on the experimental data of a single study and have not been verified using a large number of reproducible statistics. Most importantly, escape is a very rapid and transient behavior, and the limitations of detection techniques necessitate the validation of study findings. Above all, as the earliest lysosomal escape mechanism, there is a large amount of literature supporting the existence of the proton sponge effect, and we mentioned it here in the hope that there will be more experimental data to give us a clearer direction in the future, just as truth arises in debate.

#### Endosomal/lysosomal escape through osmotic lysis

pH-responsive nanoparticles can disassemble at lower pH to mediate endosomal/lysosomal escape. The disassembly of nanoparticles into numerous polymer subunits results in osmotic shock that leads to the rupture of endosomes/lysosomes (Fig. 3B) [105–107]. This process is termed osmotic lysis. Massignani et al. [55] reported that the decrease of pH in endosomes mediated the rapid disassembly of nanoparticles prepared using the deblock copolymer PMPC-bPDPAEMA into abundant monomers, and the sharply increasing osmotic pressure promoted the release of cargo. Similarly, Li et al. [56] synthesized a range of quaternary ammonium-based amphiphilic triblock polymers to assemble pH-sensitive nanoparticles. The core was hydrophobic at physiological pH; however, the nanoparticles protonated to become hydrophilic and mutually exclusive in an acidic environment. When incubated with endosomes (pH 6.5 to 6.8), the siRNA-loaded mPEG_45_-P(DPA_50_-co-DMAEMA_56_)-PT_53_ nanoparticles rapidly disassembled, leading to the cytosolic release of siRNA and enhanced gene silencing activity.

Inorganic nanoparticles, such as those prepared using Ca^2+^ [108,109] and Zn^2+^ [110], may have an enhanced endosomal/lysosomal escape ability owing to their rapid dissolution in acidic environments. Drug-encapsulated inorganic nanocarriers undergo ionic reactions and dissolve in the acidic environment of lysosomes. Subsequently, they release a large number of ions, resulting in a dramatic increase in the internal osmotic pressure of lysosomes, leading to fluctuations in lysosomal water absorption and the release of drugs into the cytosol for better effects. The most representative inorganic nanocarriers are calcium phosphate (CAP) nanoparticles. Zhang et al. [57] and Li et al. [58] constructed cationic lipid membrane-coated CAP/siRNA nanoparticles through water-in-oil microemulsion using Ca^2+^, HPO_4_^2−^, and DOPA (1,2-dioleoyl-sn-glycero-3-phosphate) as basic raw materials. Under the acidic conditions of lysosomes, the dissolution of CAP released a large number of ions, resulting in a rapid increase in lysosomal osmotic pressure, followed by water influx to disrupt lysosomal fluctuation and release siRNAs into the cytosol. These results strongly verified the lysosomal escape property of CAP nanoparticles.

**Fig. 5.**
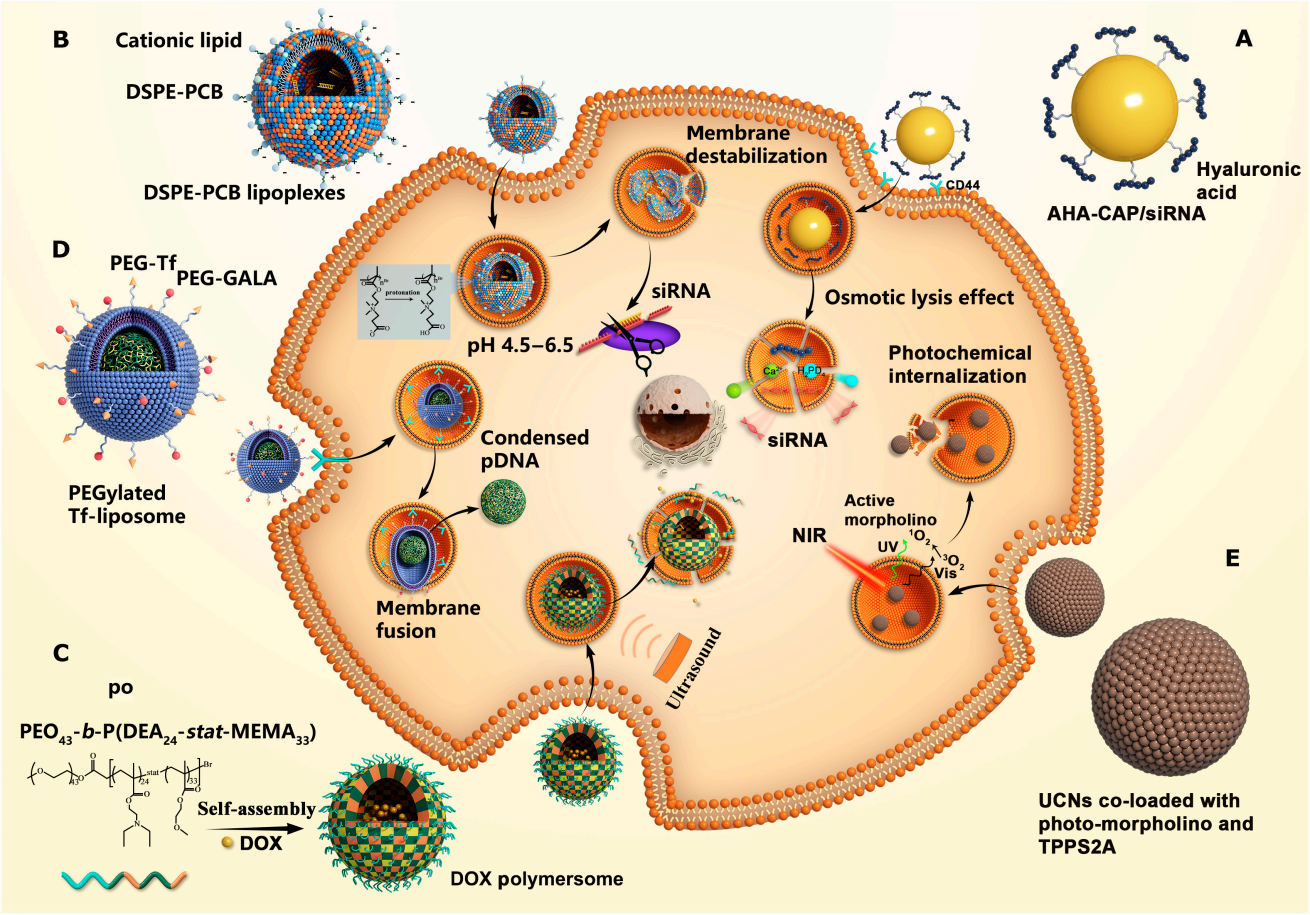
(A) Lysosomal escape induced by the pH-sensitive disassembling of CAP nanoparticles [59]. (B) Schematic diagram of DSPE-PCB lipoplexes for siRNA delivery with enhanced siRNA endosomal/lysosomal escape ability [67]. (C) Illustration of ultrasound-responsive polymersomes for facilely controlled drug delivery in tumor cells [68]. (D) GALA-mediated membrane fusion induced by the lowering of the pH in endosome and enhanced the endosomal escape of DNA core particles to the cytoplasmic space [75]. (E) The mechanism of photosensitizers enhancing endosomal escape [77]. UCNs, upconversion nanoparticles; DOX, doxorubicin; NIR, near infrared; UV, ultraviolet; Vis, visible.

CAP, a natural inorganic material with better biocompatibility and biodegradability, has high transfection efficiency and is considered a promising vehicle for gene delivery. However, its use as a nanocarrier is challenging owing to the lack of tissue specificity and the uncontrollable growth of size in a physiological solution. Therefore, various derivatives of chitosan, hyaluronic acid, and poly(ethylene glycol) (PEG) have been used to synthesize CAP nanoparticles with an enhanced lysosomal escape ability [111–117]. Qiu et al. [59] synthesized alendronate-hyaluronic acid (AHA) conjugates and prepared a novel core–shell CAP-AHA/siRNA delivery system by coating AHA around the inner core assembled through the chemical chelation of Ca^2+^ and phosphate ions (Fig. 5A). The internalized nanoparticles exhibited a pH-dependent siRNA release and contributed to rapid escape from endosomes/lysosomes. Together, nanocarriers that can promote osmotic lysis can convert the inferior environment of lysosomes into a superior environment using the acidic characteristics of lysosomes to facilitate the dissolution of nanocarriers and the release of drugs instead of only avoiding lysosomal degradation.

#### Endosomal/lysosomal escape induced by the swelling effect

Various pH-responsive materials have been introduced to design nanoparticles that can swell at a lower pH in lysosomes after endocytosis. Tang et al. [118] reported that swelling polymers can enhance transfection. They used intact and fragmented polyacrylamide (PAM) dendrimers to regulate the flexibility and pH-sensitive enlargement of nanocarriers. Compared with nanocarriers synthesized using intact dendrimers with steric constraints, those synthesized using fragmented dendrimers with optimal flexibility had higher transfection efficiency after administration. On the basis of these findings, Szoka et al. [119] advocated using the term “umbrella hypothesis” to describe the volumetric expansion of polymers during protonation. A swelling nanoparticle is similar to an open umbrella, and the rapidly expanding volume of nanocarriers disrupts the membrane and promotes lysosomal escape (Fig. 3C). In addition, the rupture of endosomes into the cytosol is partly induced by mechanical destruction owing to the swelling of nanoparticles.

Hu et al. [60] developed core–shell nanoparticles using PEG dimethacrylate (PEGDMA) as a cross-linker. The particle size was approximately 200 nm at a pH of 7.4; however, the size of the nanoparticles increased to 550 nm at a lower pH of 4.9. Between pH values of 7.0 and 6.8, rapid swelling was observed owing to the electrostatic interactions and typical solvation of charged materials in the nanoparticle core. These nanoparticles successfully released ovalbumin and the small-molecule calcine into the cytoplasm of dendritic cells. Similarly, Griset et al. [61] synthesized expansile polymeric nanoparticles to deliver paclitaxel for treating lung cancer. These nanoparticles could expand several hundred-fold in volume (from 100 to 1,000 nm) in diameter in response to pH changes in lysosomes. Similar nanoparticles have been reported in other studies, such as nebulized anionic guanidinylated O-carboxymethyl chitosan/N-2-hydroxypropyltimehyl ammonium chloride chitosan nanoparticles for pulmonary delivery of siRNAs [120], dual-layered nanogel-coated hollow lipid/polypeptide for doxorubicin delivery [62], and pH-triggered polymeric nanogel for delivering proteins to treat lysosomal storage diseases [121].

Although these particles can contribute to endosomal escape, the factors affecting swelling remain unknown [15]. First, the cross-linking density can affect the swelling and transfection efficiency of nanoparticles. Nanoparticles with a lower cross-linking density have been demonstrated to have higher transfection efficiency [122]. This phenomenon suggests that a high cross-linking density makes the nanocarriers stronger and reduces the response to pH-sensitive swelling. Villani et al. [123] presented key evidence on the effects of structure and chemical composition on the swelling of nanocarriers. In their study, A(BC)n amphiphilic block copolymers with linear (*n* = 1) and branched (*n* = 2) architectures were synthesized to obtain pH-sensitive vesicles capable of releasing drugs in acidic conditions via controlled swelling instead of disaggregation. Kermaniyan et al. [124] developed a series of pH-sensitive nanoparticles (size ranging from 85 to 100 nm) that exhibited tunable pH-induced swelling (120% to 200%) and had good buffering capacity. However, the endosomal escape ability of these nanoparticles was weak. A possible reason underlying this result may be that the nanoparticles could not break endosomes after expansion, highlighting the requirement of designing nanocarriers with a larger expansion coefficient.

#### Endosomal/lysosomal escape through membrane destabilization

Escape from endosomes/lysosomes can be achieved through membrane destabilization. This section mainly discusses various mechanisms of membrane destabilization that help to prevent the enzymatic degradation of drugs. These mechanisms include pore formation, membrane disruption, membrane fusion, and photochemical internalization.

Therapeutic drugs can diffuse from endosomal or lysosomal compartments via membrane pores created owing to the direct interaction of polymers or peptides with membranes or the self-assembled defined pores by peptides (Fig. 3D) [125]. Bacterial toxins usually consist of a transmembrane domain for inducing endosomal/lysosomal escape [126]. After the proteins or peptides present in toxins accumulate in EEs, the transmembrane domain undergoes a conformational change as the pH of endosomes decreases and inserts into the membrane of endosomes/lysosomes to form different pores: barrel stave and toroidal pores [126,127]. For example, lipid nanoparticles can enter HeLa cells and liver cells through phagocytosis triggered by rabankyrin-5 [9]. After lipid nanoparticles are encapsulated in endosomes, a pore is formed temporarily and siRNAs are released into the cytoplasm through this pore. Similarly, the HIV envelope glycoprotein gp41 can enhance endosomal escape by adopting an amphipathic α-helical structure. Therefore, modification of the cationic polymer PEI with a lytic peptide derived from the endodomain of gp41 can significantly enhance PEI-mediated siRNA delivery [64]. In addition, melittin [128] and listeriolysin O [65] are potential peptides that can result in pore formation in endosomal membranes to enhance escape.

Together, bacterial toxins and/or cargos are translocated through endosomal membranes in a folded state or released into the cytoplasm without a complete nanostructure. ur Rehman et al. [63] investigated the interaction between lipo-/polyplexes and HeLa cells. Both the polymer and its genetic payload were separately released into the cytoplasm via local pores within the endosomal membrane. Similarly, Plaza-Ga et al. [65] demonstrated that endosomal acidification led to the release of listeriolysin O (LLO) protein from the nanoparticle surface and its self-assembly into a 300-Å pore that perforated the endosomal/lysosomal membrane, enabling the escape of gold nanoparticles. However, the size of the transmembrane pores is approximately 1 to 2 nm, which limits their use in inducing an efficient release of therapeutic cargoes [129]. Recent studies have indicated that nanodiamonds with sharp corners can escape from endosomes by piercing their lipid membrane [130,131].

Various materials interact with the endosomal/lysosomal membrane mainly through electrostatic interactions and increase the instability of the membrane to promote endosomal/lysosomal escape (Fig. 3E). Owing to their natural endosomal escape property, viruses may act as potential vectors to deliver drugs; however, their high toxicity and immunogenicity hinder their application in clinical settings [132]. Cell-penetrating peptides (CPPs) are short peptides with approximately 6 to 30 amino acid residues derived from viruses [133]. The TAT peptide (RKKRRQRRR) is a typical representative of cationic CPPs that can deliver cargos to the cytosol but cannot mediate endosomal escape [134]. By contrast, the pH-responsive peptides were mostly introduced for membrane-disrupting peptides specific toward endosomal membranes because of the low pH being a trigger for membrane insertion and leakage. For example, the influenza virus can use the N-terminal fusion peptide of hemagglutinin-2 (HA-2) to destroy the endosomal membrane [135]. Under acidic conditions, protonated peptides penetrate the lipid bilayer, resulting in disruption of the membrane, and allow viral nucleic acids to effectively escape from endosomes/lysosomes to the cytoplasm [66,136]. Similarly, pH (low) insertion peptides derived from the bacteriorhodopsin protein can change their conformation into a helix under acidic conditions and penetrate the membrane for enhancing the endosome/lysosome escape ability [137]. Zhang et al. [138] demonstrated that the incorporation of pH-sensitive triple-Glu-substituted peptides (AR-23) into PLL/DNA polyplexes enhanced the disruption of endosomal/lysosomal membranes, thereby promoting their entry into the cytoplasm and increasing the transfection efficiency.

The primary mechanism underlying membrane disruption is that the charge of the material changes to positive under an acidic environment and subsequently forms ion pairs with the negatively charged endosomal membrane, thereby damaging the stability of the membrane and releasing drugs. A similar method has been used by Peng et al. [139] to develop pH-sensitive nanoparticles (Fig. 5B). They developed DSPE-PCB (polycarboxybetaine) lipoplexes using a pH-sensitive zwitterionic PCB whose negative carboxyl acid groups could be protonated at low pH [140]. Neutral PCB lipids could be protonated in endosomes/lysosomes, which promoted the fusion of cationic liposomes with endosomal/lysosomal membranes and hence enhanced the endosomal/lysosomal escape of siRNA. The timing of pH-based membrane destabilization is affected by the components of materials, which may in turn affect endosomal/lysosomal escape [141]. Cupic et al. [5] synthesized 5 pH-based disassembly pHlexi particles by combining PEG-b-PDEAEMA with random copolymers of PDPAEMA. These particles were disassembled at various pH values ranging from 7.2 to 4.9, and membrane destabilization occurred at a pH of approximately 0.5 units above the disassembly point.

**Fig. 6.**
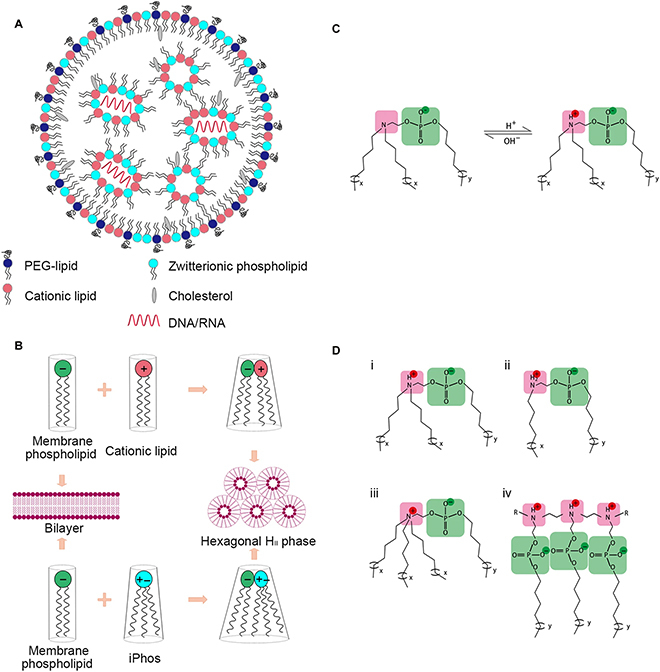
(A to D) Structure and function of designed iPhos containing ionizable amines [142].

Furthermore, ionizable lipids (pKa; 6.2) used as delivery vehicles can mediate endosomal escape (Fig. 6A) [142,143]. They become protonated and positively charged as the endosome matures (pH 6), resulting in phase transformation from a cone-shaped structure into the hexagonal HII structure (Fig. 6B and 6C), followed by localized disruption of the endosomal lipid bilayer and endosomal escape of drugs into the cytoplasm [144,145]. Studies employing cryo-transmission electron microscopy have demonstrated that the structures of newly formed lipidic assemblies highly rely on the nature of ionizable lipids [9], which often possess different numbers of hydrocarbon chain and various arrangements (Fig. 6D). The ability to escape from the endosome was explored in various lipid nanoparticles based on 3 commercially available ionizable lipids, Dlin-MC3-DMA (MC3), Dlin-KC2-DMA (KC2), and SS-OP. The result indicated that lipid nanoparticles with 1.5 mol% of PEG and a hydrocarbon chain C14 resulted in optimal endosomal escape [146]. Dong et al. [147] developed the nucleoside-modified mRNA encoding VEGFA (vascular endothelial growth factor)-encapsulated ionizable lipid nanoparticles, which indicates an excellent lysosome escape capability to improve angiogenesis and increase wound healing rate. In addition, endosomal/lysosomal escape can be accelerated by destroying the components of the membrane via cationic agents. Joris et al. [148] showed that Food and Drug Administration-approved cationic amphiphilic drugs induced the phospholipidosis of lysosomes, resulting in transient lysosomal membrane permeabilization. Sequential incubation with nanogels/siRNAs significantly enhanced gene silencing in cancer cells. Similarly, Tamura et al. [149] designed acid-degradable cationic polyrotaxanes to destabilize endosomal/lysosomal membranes via rapid removal of phospholipids from the membranes, resulting in endosomal/lysosomal escape of siRNAs for gene silencing. Additionally, Donders et al. [150] established a tunable and generalizable strategy for endosomal disruption by manipulating the pKa of colloid-forming drugs.

Furthermore, physical and mechanical characteristics can affect endosomal/lysosome escape, including ultrasound, temperature, and magnetism. Exogenous ultrasound is one of the classical membranes’ destabilizing escape methods due to the formation of transient pores under ultrasound exposure. For example, Liao et al. [151] used 2-methacryloyloxy ethyl trimethyl ammonium chloride (TMA) to prepare the TMA/plasmid DNA polyplexes, and the unique on–off phenomenon of TMA/pDNA polyplexes controlled by ultrasound exposure was found to be associated with endosomal escape. Additionally, the combination of ultrasound and nanobubbles is an important method of developing novel and noninvasive nanocarriers. Omata et al. [152,153] indicated that the transfection efficiency of TAT-PEG liposomes was enhanced by approximately 30-fold when bubble liposomes and ultrasound exposure were used to promote endosomal escape, and the mechanism was involved in endosomal acidification and vesicle fusion with Ca^2+^ and adenosine triphosphate. Similarly, Wei et al. [68] developed a new-generation ultrasound-responsive polymersome through self-assembly of PEO-b-P(DEA-stat-MEMA) block copolymers to evaluate its efficiency in delivering anticancer drugs. As shown in Fig. 5C, sonication accelerated the release of doxorubicin from the ultrasound-responsive polymersomes, resulting in rapid escape from LEs and significant inhibition of tumor growth (approximately 95%). However, these effects often depend on the amplitude of ultrasound waves used and are limited in deep tissues.

As a physical parameter, temperature can be used to enhance lysosomal escape. Alamoudi et al. [69] used PEG-modified DOTAP/DOPE/cholesterol liposomes to encapsulate both siRNA and ammonium bicarbonate to construct temperature-sensitive “bubble liposomes.” When the temperature of the tumor region was increased to 42 °C through external heating, ammonium bicarbonate was decomposed to produce carbon dioxide in the acidic environment of lysosomes, resulting in the disruption of lysosomes and effective release of Bcl2 or MRP1 siRNA into the cytoplasm.

Magnetic material-induced destabilization of lysosomes offers a novel strategy for the functionalization of nanocarriers. Superparamagnetic iron oxide nanoparticles can induce apoptosis through hyperthermia after stimulation with an alternating magnetic field [154]. Pucci et al. [155] and Domenech et al. [156] designed lipid-based nanocarriers encapsulating superparamagnetic iron oxide nanoparticles that resulted in membrane permeabilization with the consequent release of cargo from lysosomes.

Membrane fusion refers to the process through which 2 closely attached lipid membranes merge into a bilayer. It is a potential strategy for overcoming the poor release of nanodrugs from endosomes. The anionic lipids of endosomes interact with the cationic lipids of nanocarriers and rearrange to form a neutral ion pair, which sharply destabilizes the membrane and enhances endosomal escape (Fig. 3F).

Owing to its lamellar structure, liposomes can fuse more easily with lipid components present on the endosomal/lysosomal membranes to promote escape [157]. Dioleoyl-phosphatidyl ethanol-amine (DOPE) is the most commonly used membrane fusion agent (helper lipid) in delivery systems [158–160]. It can form hexagonal crystals under acidic conditions to induce membrane fusion between liposomes and endosomes or lysosomes [161,162]. The cationic lipid 1,2-dioleoyl-3-trimethylammoniumpropane (DOTAP), DOPE, and other helper lipids were used to prepare a series of siRNA-loaded core–shell nanoparticles for the treatment of breast cancer [70,71] and brain cancer [72,73]. After 2 h of incubation, the concentration of siRNAs was high in the cytoplasm than in lysosomes because DOPE as the helper lipid facilitated the fusion of liposomes with the lysosomal membrane through formation of a hexagonal inverted phase (HII) structure, thus allowing the efficient escape of nanocarriers into the cytosol.

Various pH-sensitive peptides can induce membrane fusion and enhance the endosomal/lysosomal escape of nanodrugs. Wyman et al. [163] designed the KALA peptide, whose amino acid sequence is WEAKLAKALAKALAKHLAKALAKALKACEA, and linked it with DNA to destroy the stability of the membrane. The conformation of KALA changes with pH, and KALA facilitated the escape of DNA from lysosomes under acidic conditions. Shaheen et al. [74] designed multilayer nanoparticles modified with octa arginine, in which KALA was decorated on the outermost layer of nanoparticles and had lysosomal escape ability. The transfection efficiency of nanoparticles modified with the functional KALA polypeptide was 20 times higher than that of unmodified nanoparticles.

Similarly, the GALA peptide, a 30-residue artificial amphiphilic peptide having a repeated sequence of Glu-Ala-Leu-Ala, can convert its structure to a helix (an amphipathic α-helical structure) when the pH is reduced from 7.0 to 5.0 [164]. The helical structure has a high affinity for the negatively charged endosomal/lysosomal membrane and forms aqueous pores consisting of 10 ± 2 peptides with a head-to-tail (N- to C-terminus) orientation [165,166]. Sasaki et al. [75] developed a novel method for encapsulating condensed plasmid DNA into PEGylated Tf-liposomes (Tf-PEG-L) to form core–shell-type nanoparticles (Fig. 5D). The transfection efficiency increased almost 100-fold because GALA interacted synergistically to induce the fusion of liposomes and endosomes/lysosomes.

Photochemical internalization is a light-triggered novel technology initially developed at the Norwegian Radium Hospital to promote endosomal/lysosomal escape of various nanocarriers encapsulated with therapeutic agents [11,76,167]. This unique mechanism relies on the activation of light to disturb an endocytic structure, usually exploits a small molecule that is susceptible to light and can help to produce a large amount of oxidizing active substances after illumination to disrupt the endosomal/lysosomal membrane for the release of encapsulated drugs (Fig. 3G).

Naturally, photosensitizers that form triplet states and can produce reactive oxygen species have tricyclic, heterocyclic, or porphyrin-like ring structures with conjugated double bonds (π-electron system), including disulfonated meso-tetraphenylporphine (TPPS2A), phthalocyanine dendrimers, disulfonated aluminum phthalocyanine, and tetra(4-sulfonatophenyl) porphine [168,169]. Jayakumar et al. [77] created silica core–shell nanoparticles that could emit both ultraviolet and visible light to active TPPS2A for enhanced gene knockdown (Fig. 5E). Cabral et al. [78] developed a novel nanocarrier using thiolated camptothecin and thiolated PEG-b-poly(glutamic acid) for photoactivated targeted chemotherapy. The results indicated that photochemical internalization-induced endosomal escape triggered drug release (approximately 90%) after 24 h.

Together, it is important to consider the influence of materials on membrane stability when designing nanocarriers. Compared with membrane disruption and pore formation, membrane fusion promotes drug delivery to the cytoplasm irrespective of the particle size [106]. Given that lysosomes play a key role in regulating apoptosis, the potentially toxic effects of endosomal escape should be considered to avoid the uncontrolled release of cathepsin, which significantly affects the viability of cells [170,171].

### Directly crossing the cell membrane to bypass endosomes/lysosomes

Directly penetrating the membrane and entering the cytoplasm could bypass the degradation and destruction of endosomes/lysosomes, which is undoubtedly a good choice for the subsequent effects of drugs. Actually, for some nanocarriers, the cellular uptake mechanism is fusion with the cell membrane and entering into the cytoplasm directly without endocytosis (Fig. 2B).

**Fig. 7.**
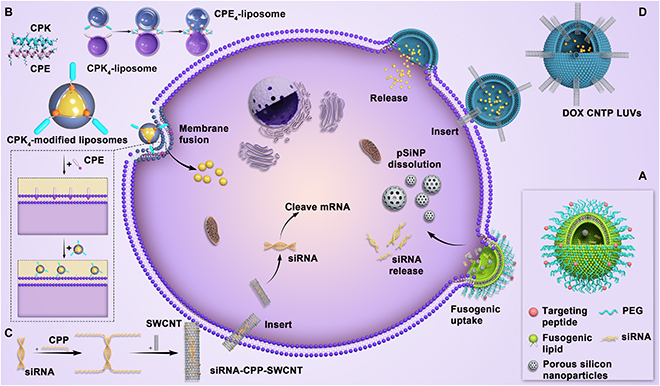
(A) Schematic of the fusogenic porous silicon nanoparticles structure and hypothesized uptake for direct delivery to target cell [79]. (B) Schematic representation of the coiled-coil structure between peptides E and K, targeted liposome fusion mediated by the coiled-coil formation between CPE_4_-modified liposomes and CPK_4_-modified liposomes, and scheme of fusion between cells and liposomes [81]. (C and D) The membrane fusion induced by the carbon nanotube [83,84]. pSiNP, porous silicon nanoparticles;LUVs, large unilamellar 1,2-dioleoyl-sn-glycero-3-phosphocholine (DOPC) vesicles; SWCNT, single-wall carbon nanotube; CNTP, carbon nanotube porin.

Researchers have designed several delivery systems based on this strategy, mainly focusing on proteins, peptides, or other biomaterials. This endosomal/lysosomal evading mechanism is especially observed in virus-infected cells. Kim et al. [79] designed siRNA nanocarriers using porous silicon nanoparticles functionalized with tumor-targeting peptides and fusogenic lipids (Fig. 7A). The lipid coatings induced membrane fusion and promoted entry into cells through a distinctive mechanism that is independent of receptor-mediated endocytosis. Similarly, Yang et al. [43] prepared fusogenic exosomes by modifying exosomes with vascular stomatitis virus fusion protein. These modified exosomes could fuse with the plasma membranes through a process called “membrane editing,” which facilitated the transfer of biologically active membrane proteins into target cell membranes both in vitro and in vivo and significantly improved the transfection efficiency. This mechanism also applies to the binding and fusion of other extracellular vesicles [172].

The cell membrane has unique biological properties. Cell membrane-coated nanoparticles have the complex and unique surface physicochemical properties of protocells, especially the ability to fuse with nanocarriers [173]. Therefore, cell membrane-coated nanoparticles, primarily those coated with blood, bacterial, and tumor cells, have attracted extensive attention from researchers [174]. These nanoparticles can be prepared using the following method: Cells are lysed in a hypotonic solution, and the cell membrane is purified through differential centrifugation. The cell membrane is extruded from the polycarbonate membrane (aperture, 200 to 400 nm) several times to obtain cell membrane fragments with a uniform particle size. Subsequently, the cell membrane is coated or fused on the nanocore surface via repeated extrusion or ultrasonication. The nanocores are prepared using hard organic nanoparticles (such as PLGA (poly(lactic-co-glycolic acid) and PCL nanoparticles) or inorganic nanoparticles (gold nanoparticles, carbon nanorods, silica nanoparticles, and quantum dots) [175–177].

Red blood cells have several surface proteins that play a key role in recognizing homologous red blood cells. Guo et al. [178] proposed a biomimetic and controlled route to achieve the fusion of hydrophobic quantum dots with red blood cell membranes, and the resulting red blood cell-encapsulated quantum dots could fuse with the cell membrane. Similarly, Xuan et al. [179] coated leukocyte membranes on the surface of silica nanoparticles to synthesize a leucocyte-like carrier for targeted treatment of lesions. Wang et al. [180] used leukocyte membranes activated by inflammatory factors to construct “grapefruit-derived” nanocarriers. The experimental results revealed that doxorubicin-encapsulated nanoparticles effectively inhibited tumor growth, whereas curcumin-encapsulated nanoparticles effectively alleviated colitis. In addition, considering the hemostatic mechanism and function of platelets, researchers have developed nanocarriers modified with platelet membranes. For example, Anselmo et al. [181] coated the surface of nanoparticles with platelet membranes and prepared platelet-like nanocarriers, which specifically aggregated at the injured site and promoted coagulation. Surface antigens, such as galactoagglutinin-3 and carcinoembryonic antigen [182], specifically expressed by tumor cells have a structural domain that allows adhesion to homologous cells and, hence, endow nanoparticles with a unique tumor-targeting ability. Sun et al. [80] used PCL and F68 to encapsulate PTX to obtain core nanoparticles and coated them with the membrane of 4T1 tumor cells to obtain integrated CPPNs (cancer cell membrane-coated paclitaxel-loaded polymeric nanoparticles). These nanoparticles not only effectively inhibited the growth of tumors in situ but also significantly inhibited the metastasis of 4T1 tumors.

Cell-penetrating peptides (CPPs) have been used in the construction of vectors owing to their unique membrane-penetrating function. For example, polyarginine (R4 to R9) promotes membrane fusion not by direct penetration [17] but by “punching” the target membrane, resulting in the direct release of cargo into the cytoplasm. Jiang et al. [183] constructed nanoparticle-stabilized nanocapsules (NPSCs) to achieve direct delivery of siRNAs into the cytosol. siRNAs were allowed to adsorb on arginine-modified gold nanoparticles through electrostatic interactions, followed by the self-assembly of fatty acid nanodroplets on the surface to construct NPSC/siRNA nanocapsules, which could rapidly deliver siRNAs to the cytosol via membrane fusion. Similarly, Deng et al. [184] coated a tetrapeptide on the surface of “MEND” carriers delivering pDNA and found that it had no evident colocalization with lysosomes. The carrier entered the cell through direct membrane fusion, which was named “one-step into the cytoplasm”; however, the specific underlying mechanism was not discussed. Moreover, whether the nanocarrier was transported through mechanisms other than membrane fusion remained uncertain. To this end, Yao et al. [185] reported a comprehensive mechanism. They coated a tumor microenvironmental pH-sensitive polypeptide on the surface of PEG liposomes. In the tumor microenvironment, the polypeptide was exposed responsively and fused with lipid molecules on the cell membrane, as well as improved the uptake effect by directly fusing and releasing drugs to the cytoplasm or promoting lysosome escape after endocytosis. This membrane fusion method was very rapid and only consumed 8 ms. However, the carrier was not completely inserted into the cell directly; a large part of the carrier was endocytosed and transferred through lysosomes.

Lipid membrane fusion is the basic characteristic of cells to maintain efficient activity, especially during endocytosis, exocytosis, and intracellular transport [186–189]. However, it often requires a condition that the carrier contains ligand A, and the cell membrane surface has its receptor B. A and B interact to induce membrane fusion when they approach each other at a nanometer distance. For example, membrane fusion is mediated by SNAREs, which are membrane proteins whose C-terminal tail is anchored at the membrane. SNAREs are widely distributed in the ER, Golgi apparatus, and endosomes and sparsely distributed on the cell membrane [157,190]. According to the SNARE hypothesis, v-SNARE on transport vesicles (v represents the vesicle) specifically interacts with homologous t-SNARE on the target membrane (t represents the target). t-SNARE is usually complex composed of 3 SNARE motifs, which are composed of 3 Syn or Syn-like SNARE motifs, whereas v-SNARE usually contains one SNARE motif. During v-t-SNARE pairing, the formation of a stable 4-helix bundle induces the fusion of the 2 diaphragms. Ding et al. [191] demonstrated that stably assembled rHDL/Chol–siRNA complexes crossed the membrane and directly entered the cytoplasm via the scavenger receptor BI (SR-BI)-mediated non-endocytic mechanism, thereby evading endosomes/lysosomes. This phenomenon was observed in tumor cells with high expression of SR-BI, such as human breast cancer MCF-7 cells and human hepatocellular carcinoma HepG2 cells, but not in those with low expression of SR-BI, such as human fibrosarcoma HT1080 cells, indicating a selective behavior because of the interaction between A and B described previously.

Notably, not all cell surfaces contain corresponding receptors for membrane fusion. As shown in Fig. 7B, Yang et al. [81] designed a pair of complementary coiled-coil lipopeptides (CPK (cholesterol-PEG-peptides (KIAALKE)_4_) and CPE (cholesterol-PEG-peptides (EIAALEK)_4_)) and embedded them in the lipid bilayer of liposomes and cell membranes, respectively. When CPK-decorated liposomes were close to CPE-modified cell membranes, they interacted and induced membrane fusion with the concomitant release of liposome-encapsulated cargo for direct drug delivery without endocytosis. Similarly, Zhou et al. [82] developed an oligonucleotide (ON) template-assisted polymerization approach to synthesize ON nanospheres as gene vectors. In this approach, guanidinium-containing disulfide monomers were organized on templates, greatly increasing their local effective concentrations. Consequently, ring-opening disulfide-exchange polymerization between monomers was accelerated, further facilitating the self-assembly of ON nanospheres. These nanospheres were directly delivered into the cytosol via an endocytosis-independent pathway, followed by intracellular depolymerization in the reductive cytosolic environment to release the cargo, resulting in efficient gene silencing.

Introducing an intermediate material into nanocarriers for membrane fusion, such as carbon nanotubes [192], is a strategy for adjusting the mode of membrane penetration. Jiang et al. [83] designed the nanoplexes by encapsulating siRNAs with CPPs (NH_2_-VGalAvVvWlWlWlWbA-GSG-PKKRKVC-COOH). The siRNA–CPP complex was transported to lysosomes through endocytosis, whereas the siRNA–CPP complex modified with single-wall carbon nanotubes directly penetrated the cell membrane and had weak colocalization with lysosomes (Fig. 7C). Similarly, Ho et al. [84] reported that liposomes studded with 0.8-nm-wide carbon nanotube porins functioned as efficient vehicles for direct cytoplasmic drug delivery by facilitating the fusion of lipid membranes and complete mixing of the membrane material and vesicle content (Fig. 7D). Molecular dynamics simulations indicated that short fragments of carbon nanotubes inserted into lipid membranes potentially facilitated the fusion of lipid membranes [193,194]. In addition, Tai and Gao [195] reported the development of small, bifunctional chemical tags capable of transporting siRNAs directly into the cytosol. The bifunctional tags consisted of a siRNA-binding moiety that interacted with siRNAs noncovalently and a steroid domain that readily fused with the mammalian cell membrane. Compared with the conventional covalently conjugated siRNA–steroid complex that entered cells largely via endocytosis, which substantially limited siRNA bioavailability, the noncovalently tagged siRNAs directly penetrated the cell membrane, avoiding endocytosis.

As a strategy for overcoming lysosomal barriers, direct penetration into the cell membrane can prevent lysosomal degradation to some extent, but has strong limitations owing to the potential cytotoxicity of inorganic materials. Moreover, the transport mechanism remains elusive, thus limiting the application of membrane fusion. Direct penetration into the cell membrane is an interesting approach; however, it is challenging because delivery systems enter different cells nonspecifically.

### Intracellular transport pathway bypassing endosomes/lysosomes

Unlike the endosome–lysosome pathway, the endosome–Golgi/ER pathway is considered a non-degradative pathway (Fig. 2C). Some studies have demonstrated that nanoparticles entering cells through macrocytosis or caveolin-mediated endocytosis are partly transported through the endosome–Golgi/ER pathway [46,196,197]. Small-molecule substances, toxins [198], or siRNAs [85] are internalized by cells through neutral vesicles called caveosomes, bypassing the endosomal/lysosomal pathway, and are directly transported to the Golgi apparatus or ER [86,87]. Reilly et al. [86] demonstrated that histone-targeted polyplexes were transported to the nucleus via the endosome–Golgi/ER retrograde pathway, evading endosomal/lysosomal trafficking routes. This transport mechanism was mainly induced by DPY-30, which was located in TGN [199].

However, some researchers have questioned the “caveosome-related pathway.” Owing to the identification of EEA-1, a maker protein of EEs, on caveosomes, researchers have indicated that the so-called caveosome is an EE, denying the existence of caveosome [200,201]. In addition, studies have reported that some nanoparticles are transported to lysosomes via caveolin-mediated endocytosis [202]. We speculate that the caveosome does not exist; however, the way in which nanoparticles enter cells should be closely related to subsequent intracellular localization. Different cell types, nanocarriers, and concentrations affect the underlying mechanism, and this relationship cannot be directly denied because of the cellular uptake mechanism and location on a certain cell.

Moreover, changing the formulation and synthesis process may regulate the intracellular transport pathway of nanoparticles, providing a new method for avoiding lysosomal degradation. We have previously investigated the lysosomal localization of nanocarriers with different assembly structures of gemini-like cationic lipid (CLD)/siRNA prepared using the AT (siRNA solution mixed with preformed CLD NPs), MT (ethanolic CLD solution dropped in a solutionof siRNA under sonication), and HT (a CLD thin film hydrated with siRNA solution) methods [203,204]. The siRNA nanoparticles prepared using the MT method mainly entered the cells through macrocytosis and caveolin-mediated endocytosis and did not colocalize with lysosomes.

**Fig. 8.**
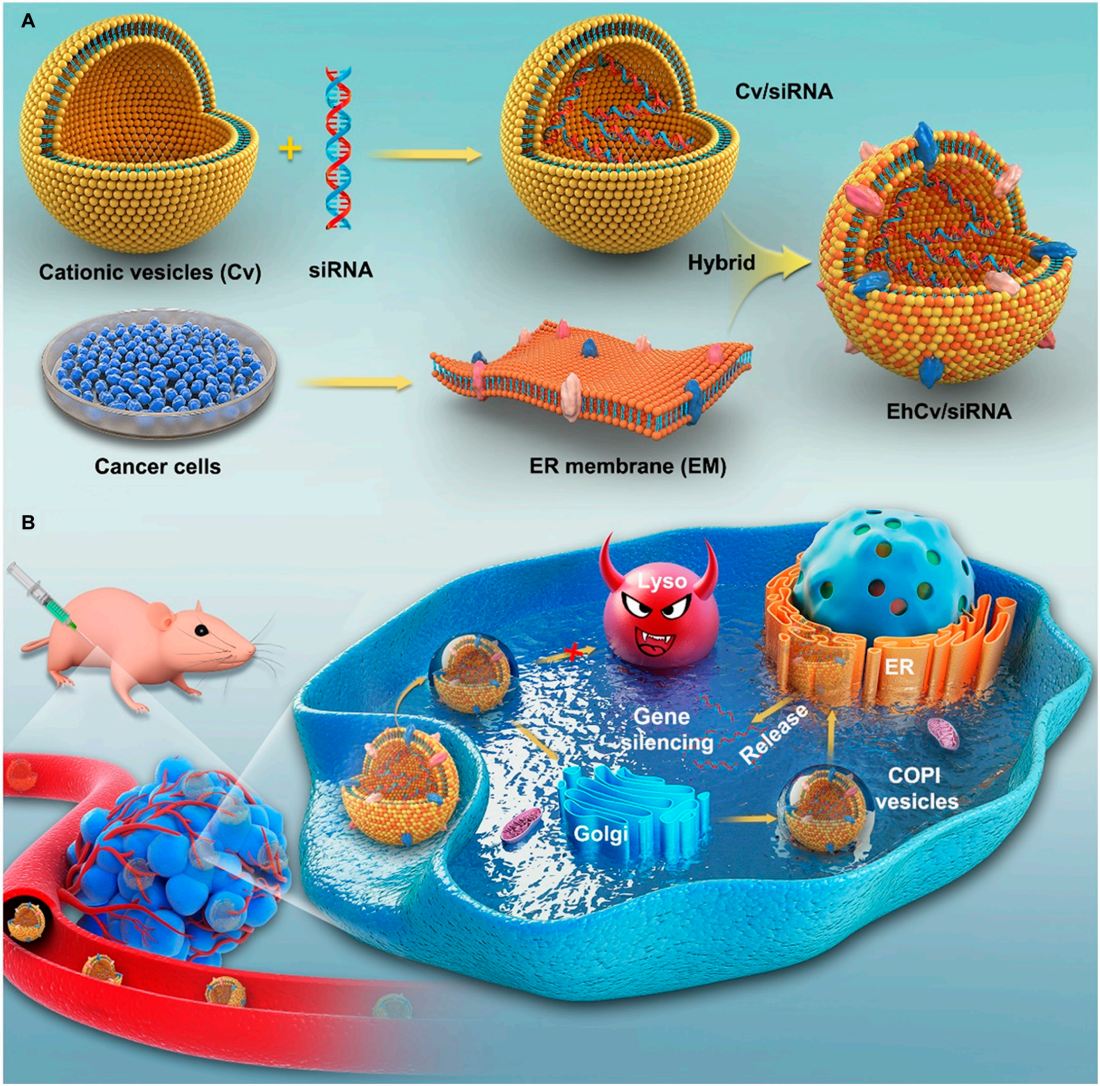
(A and B) Schematic of siRNA-loaded nanoplexes evading lysosomal degradation via an ER membrane-modified strategy [89].

Furthermore, the intracellular behavior of nanoparticles can be regulated through the functional modification of carriers by mimicking biochemical properties. Wang et al. [88] used the resident KDEL peptide of ER membrane proteins to modify gold nanoparticles containing siRNAs and found that the nanoparticles were internalized via CME and exhibited strong colocalization with the ER. KDEL can mediate retrograde transport from the Golgi apparatus to ER via COPI vesicles and helps co-transported drugs evade the lysosomal degradation pathway. The reverse transport of endosomes to Golgi or Golgi to ER may result in retrograde transport. The ER membrane derived from cancer cells has been used to prepare hybrid nanoplexes to enhance siRNA transfection (Fig. 8) [89]. Functional proteins on the ER membrane can alter the intracellular trafficking pathway of endosome–Golgi–ER to evade lysosomal degradation. However, it is noteworthy that different sources of the ER membrane and the combination of components can lead to uncertainty in formulation components and necessitate further simplification of formulation to ensure batch repeatability.

## Conclusion and Prospects

After decades of development and innovation, the use of nanotechnology-based drug therapies has increased in clinical practice in recent years as evidenced by the increasing number of clinically approved nanodrugs annually. Nanocarriers can penetrate biological barriers, improve biocompatibility, prolong the circulation time of drugs, and achieve targeted delivery of drugs. However, the lack of a safe and effective nanodrug delivery system limits the clinical application of nanodrugs. Moreover, the use of nanodrugs is limited owing to their immunogenicity, off-target effects, toxicity, and low stability. Low delivery efficiency owing to endosomal/lysosomal entrapment is a key problem that should be addressed.

Therefore, a comprehensive understanding of the mechanisms of escape from endosomes/lysosomes is important for enhancing the transport efficiency of nanocarriers. This review summarized recent advances in the development of different nanocarriers and described strategies for overcoming endosomal/lysosomal barriers based on the perspective of cellular uptake and intracellular transport mechanisms. The 3 main strategies are as follows: (a) strategies promoting the escape of endosomes/lysosomes, (b) strategies promoting non-endocytosis delivery through direct penetration into the cell membrane to evade endosomes/lysosomes, and (c) strategies involving a detour pathway to bypass lysosomes and evade degradation. Four advanced strategies for promoting escape from endosomes/lysosomes were discussed in this review, including the proton sponge effect of pH buffering; osmotic lysis resulting from pH-responsive disassembly of nanoparticles; as well as swelling of pH-responsive nanoparticles and membrane destabilization induced by pore formation, membrane disruption, membrane fusion, and photochemical internalization. Each nanocarrier has a unique intracellular fate owing to different physicochemical properties.

Traditional Chinese culture propagates a theory of yin and yang, which signifies that there is no absolute good or bad and everything is balanced. As acidic and enzyme-rich organelles, lysosomes play a crucial role in maintaining cell function. We speculate that strategies involving direct penetration into the cell membrane and evasion of lysosomes through a detour pathway are more promising for nanodrug delivery. However, some nanocarriers may benefit from lysosomal involvement to a certain degree, such as CAP nanoparticles that disassemble in response to the pH of lysosomes and liposomes that reverse surface charges under the acidic conditions of lysosomes to release drugs into the cytoplasm with better efficiency. The most important aspect of drug delivery is the reasonable design of carriers according to the requirements so that lysosomes can be used as a helper instead of a barrier.

Importantly, these advances may be more complex because the mechanism of trafficking, and escape depends on the characteristics of vehicles and cell types. Existing strategies for overcoming endosomal/lysosomal barriers may increase the delivery efficiency of nanocarriers; however, various challenges should be considered while designing nanocarriers in the future, including safety and biocompatibility, scalability and manufacturing, regulatory considerations, and so on. Liposomes are currently the main nanodrugs used in clinical practice or in clinical trials because of their good safety and biocompatibility. Importantly, the structure of liposome is stable, and it maintains high consistency of activity during the expansion from small to large batch preparation. Hence, various strategies based on liposomes to overcome endosomal/lysosomal barrier will be more potential, such as introducing cell membrane, ER membrane proteins, or DOPE into liposomes. Meanwhile, the translation of these strategies to clinical settings will require scalable and cost-effective manufacturing processes, and it is important to provide a standardized protocol to ensure reproducibility and quality control, as well as consider regulatory requirements based on their safety, efficacy, and quality.

Moreover, interdisciplinary collaboration is a crucial manner to unlock the smarter nanocarriers with innovative strategies for overcoming lysosomal barriers. Given the wide range of disciplines involved in nano-delivery systems, this includes collaborations between researchers from the fields of chemistry, materials science, biology, immunology, and medicine, as well as partnerships with industry and regulatory agencies. Finally, an in-depth understanding of nanocarrier trafficking may facilitate the rational design of smarter nanodrug delivery systems.
